# Population and colony structure and morphometrics in the queen dimorphic little black ant, *Monomorium* sp. AZ-02, with a review of queen phenotypes in the genus *Monomorium*

**DOI:** 10.1371/journal.pone.0180595

**Published:** 2017-07-17

**Authors:** Robert A. Johnson, Rick P. Overson

**Affiliations:** 1 School of Life Sciences, Arizona State University, Tempe, Arizona, United States of America; 2 Plant Conservation Science Center, Chicago Botanic Garden, Glencoe, Illinois, United States of America; University of North Carolina at Greensboro, UNITED STATES

## Abstract

The North American little black ant, *Monomorium* sp. AZ-02 (subfamily Myrmicinae), displays a dimorphism that consists of alate (winged) and ergatoid (wingless) queens. Surveys at our field site in southcentral Arizona, USA, demonstrated that only one queen phenotype (alate or ergatoid) occurred in each colony during the season in which reproductive sexuals were produced. A morphometric analysis demonstrated that ergatoid queens retained all specialized anatomical features of alate queens (except for wings), and that they were significantly smaller and had a lower mass than alate queens. Using eight morphological characters, a discriminant analysis correctly categorized all queens (40 of 40) of both phenotypes. A molecular phylogeny using 420 base pairs of the mitochondrial gene *cytochrome oxidase I* demonstrated that alate and ergatoid queens are two alternative phenotypes within the species; both phenotypes were intermixed on our phylogeny, and both phenotypes often displayed the same haplotype. A survey of the genus *Monomorium* (358 species) found that wingless queens (ergatoid queens, brachypterous queens) occur in 42 of 137 species (30.6%) in which the queen has been described. These wingless queen species are geographically and taxonomically widespread as they occur on several continents and in eight species groups, suggesting that winglessness probably arose independently on many occasions in the genus.

## Introduction

Dispersal polymorphisms have played a significant role in our understanding of population dynamics, dispersal dynamics, life history, and the physiological and biochemical basis of adaptation [1, 2]. Such polymorphisms are common among insects, and typically consist of one form that is fully capable of flight and a second form that is flightless. At the proximate level, these dimorphisms result from variation in genotype, environment, or from a combination of both; at the ultimate level, flight ability is associated with habitat persistence. Flight allows organisms to disperse from heterogeneous or temporary habitats, while flightlessness promotes survival in homogeneous or persistent habitats [2–5].

Within ants, reproductive females (queens) exhibit two types of dispersal polymorphisms. One type involves a size dimorphism in which both queen forms possess wings and are capable of flight, i.e., macrogynes and microgynes (for a review, see [6]). The second type of dimorphism involves presence or absence of wings, i.e., winged and ergatoid (= wingless) queens. In both cases, the two queen forms typically exhibit different colony founding strategies. Macrogynes and winged queens usually fly from colonies to mate and found colonies independently, whereas their small-bodied (microgynes or ergatoid queens) counterparts have limited ability to disperse and employ dependent colony founding [6–9]. Additionally, queens of some ant species have fully-developed, functional wings, but they do not fly (see [8]), indicating that lack of dispersal can result from behavioral decisions even when the queen can fly.

The ant genus *Monomorium* (subfamily Myrmicinae) is ideal for examining evolution and development of queen phenotypes because species with ergatoid and brachypterous (short, non-functional wings) queens, along with or in place of fully winged (alate) queens, are relatively common. This was emphasized by Wheeler [10], who said *Monomorium* is the best genus in which to trace the transitions in thoracic and wing structure from that of the queen to that of the worker. Despite this pronouncement, little is known about natural history, morphology, or development for species of *Monomorium* (but see [11–14]).

This paper was motivated by observations that winged (alate, or dealate after the wings are shed) and ergatoid queens co-occur in *Monomorium* sp. AZ-02. This study extends our understanding of queen dimorphism for ants in general and this species in particular by: (1) conducting a morphometric analysis of alate and ergatoid queens, (2) determining if alate and ergatoid queens belong to the same gene pool, (3) assessing for type of reproductive queens produced in colonies, and (4) providing observations of mating flights in this species. We also survey *Monomorium* to determine species that have ergatoid queens, brachypterous queens, or worker-queen intercastes/intergrades.

## Methods

### Study site

We studied *Monomorium* sp. AZ-02 at Coon Bluff in the Salt River Recreation Area, Maricopa County, Arizona, USA (33°33’N, 111°39’W; elevation 410 m). Habitat at the site consisted of small to large stands of velvet mesquite (*Prosopis velutina*) separated by open Sonoran Desert habitat that consisted of scattered creosote-bush (*Larrea tridentata*) and triangle-leaf bursage (*Ambrosia deltoidea*). Other common ants at the site included *Pogonomyrmex rugosus*, *Solenopsis xyloni*, and *Dorymyrmex insanus*.

### Nest excavations

Nests were located during July and August by baiting foragers with powdered animal crackers; foragers were followed to the nest and the entrance was marked with a wire flag. All colonies were located in partial to fully shaded microsites beneath the canopy of trees. The nest entrance consisted of a minute hole that was only slightly wider than the body of workers; small amounts of litter often surrounded or covered the nest entrance. A 10–15 cm diameter crater was excavated around each nest entrance, followed by slowly pouring 1.0–1.5 l of water into the crater. This process insured that water infiltrated into the nest area. Most species of desert ants, including *Monomorium* sp. AZ-02, have mating flights that are triggered by summer rainfall, and this technique triggers movement of workers and sexuals to the top of the nest, where they are more easily excavated. Colonies were partially excavated after 24–48 h to assess the phenotype of new, virgin queens produced by each colony. All queens were collected and returned to the laboratory, and each queen was identified (alate or ergatoid) and counted under a binocular microscope. A total of 43 colonies were excavated that contained more than one queen.

### Morphometric and mass comparisons between alate and ergatoid queens

We measured 10 external characters to assess morphometric differences between alate and ergatoid queens of *M*. sp. AZ-02. These characters included head width, head length, maximum eye diameter, scape length, mesosoma width, mesosoma length, petiole width, postpetiole width, hind femur length, and width of the first gastral tergum. Characters were measured by projecting an image from a binocular microscope to a video monitor; the image on the monitor was measured using ImageJ (available at http://rsb.info.nih.gov/nih-image/). Measurements were calibrated using photographs of an ocular micrometer scaled in 0.1 mm increments. Characters were measured on 20 alate queens and 20 ergatoid queens. In each case, measurements were taken on a maximum of two queens per colony.

We performed a morphometric comparison between alate and ergatoid queens by including the ten characters in a multivariate analysis-of-variance (MANOVA). An *a posteriori* univariate F test (tests of between-subjects differences) was used to determine which variables contributed to overall differences [15]. Degree of overlap between the two phenotypes was then assessed by performing a discriminant analysis using characters that differed significantly between the two phenotypes. The discriminant analysis developed predictive discriminant functions for each phenotype, which were applied to all queens during the same execution of the model [15]. Characters were entered into the model simultaneously using phenotype as the grouping variable. The model used *a priori* classification and equal prior probabilities.

Queen to worker body mass ratio is associated with method of colony founding [16]. Consequently, we also measured dry mass of queens and workers, then calculated queen to worker mass ratios by collecting five to ten queens and workers from four colonies bearing queens of each phenotype. Individuals were placed in a drying oven at 50–55° C for >72 h, then weighed to 0.001 mg on a Mettler MX5 microbalance. Dry mass of queens and workers were averaged within each colony, and these mean colony values were used to calculate the grand mean for each phenotype. Likewise, the queen to worker mass ratio (queen mass/worker mass) was calculated for each colony, then averaged across colonies. Average mass of each phenotype and queen to worker mass ratios were compared using a t-test. Alate and ergatoid queens were also dissected to determine presence or absence of flight muscles and phragmata.

### Lineage analysis of alate and ergatoid queens

Workers in the *M*. *minimum*-group have few diagnostic characters such that they are very difficult to identify to species (see also [17]). Consequently, we used mitochondrial gene sequences to assess whether alate and ergatoid queens belong to the same gene pool, i.e., the same species, and if the two alternative phenotypes represented one intermixed genetic lineage or two genetic lineages. In the latter case, the two phenotypes would consist of separate clades within the species. For mitochondrial sequences, we used the LEP-F1, LEP-R1 primer pair [18] to amplify a 420 base pair sequence of the *cytochrome c oxidase 1* (CO1) for each sample. Sequences were aligned using MUSCLE version 3.8.31 [19] within the program AliView [20]. Each base in the alignment was manually assessed for quality, and nucleotide ambiguity codes were added where necessary. Our phylogeny included one alate queen from each of seven colonies and one ergatoid queen from each of seven colonies. We also included one alate queen of *M*. sp. AZ-02 from south of Tucson, AZ (≈ 200 km south of our study site), two additional *M*. *minimum*-group species (*emarginatum* [Maine: Kennebec County, S.P. Cover #7074]; *viridum* [NY: Suffolk County, S.P. Cover #8671]), and one species that is outside of the *M*. *minimum* species-group (*M*. *floricola* [FL: Highlands County, M. Deyrup #5300]).

A neighbor-joining tree was estimated according to Saitou and Nei [21] with distance derived by Kimura 2-parameter pairwise distance [22] using the software R [23] and the APE library [24]. Branch support was calculated from 10,000 bootstrap replicates; branches with <50% bootstrap support were collapsed. Within-queen phenotype and between-queen phenotype differences in sequence divergence were compared using R by performing pairwise Mann-Whitney-Wilcoxon tests on Kimura 2-parameter distances for queens from Coon Bluff [25]. All sequences are deposited in Genbank: KY500952–KY500969. Voucher specimens were deposited at MCZ (Harvard University, USA) and the collection of Robert A. Johnson, Tempe, AZ, USA.

### Survey of ergatoid and brachypterous queens in *Monomorium*

We used AntCat (http://www.antcat.org/) and a catalogue of *Monomorium* (provided by B. Bolton) to obtain a list of *Monomorium* species in which the queen has been described. Queen descriptions were then obtained from publications on Antwiki (http://www.antwiki.org/wiki/World_Ant_Taxonomists).

We use the term ergatoid to denote permanently wingless queens, which also includes those referred to as apterous. Brachypterous denotes queens with short, non-functional wings, and it includes those referred to as subapterous. For each species, the queen was assumed to be fully alate unless the description specifically indicated that the queen was ergatoid, wingless, apterous, or brachypterous. Queens of several species were described without mention to wings or a dealate queen; queens were assumed to be fully alate in these cases. For at least two species, the phenotype of undescribed queens is mentioned anecdotally in the literature (e.g., *biroi* and *damarense* [11, 26]).

Species known to have worker-queen intercastes or worker-queen intergrades (WQI) also were included because: (1) WQI are common in *Monomorium*, and their reproductive status has not been established (see [27]), and (2) in the queen description under general characters for each species, Heterick [28, 29] stated that he examined ergatoid queens or WQI. Thus, it is unclear what caste he examined because he combined ergatoid queens and WFI into one category. We also include one species (*M*. *falcatum* = *Schizopelta falcata*) [30] where the caste was described as “pseudogyne”. The description given in McAreavey [30] appears to be that of a WQI (C. Peeters, pers. comm.), and we include it for the reasons stated above. These species were not included in our estimate of the percentage of species with ergatoid or brachypterous queens. Rather, they were included to emphasize that the queen phenotypes of these species need to be examined in more detail.

This study did not involve endangered or protected species. A collecting permit was obtained from the Tonto National Forest.

## Results

### Morphometric comparison and mass ratios

Alate queens of *M*. sp. AZ-02 were significantly larger than ergatoid queens (Wilks’ λ = 0.049, *F*_10, 29_ = 56.6, *P* << 0.001). Eight of the 10 characters (all but head length and scape length) were significantly greater for alate queens based on tests of between-subjects differences (univariate *F* tests within MANOVA, *P* < 0.001; Table 1).

**Table 1:** Morphological measures (means ± 1 SE; values in mm) for alate and ergatoid queens of *Monomorium* sp. AZ-02; *N* = 20 per caste (≤ 2 queens per colony).

	Queen phenotype	
Character	Alate queen	Ergatoid queen
**Head**		
** Head width (HW)**	**0.62** ± **0.00**	**0.60** ± 0.00*
** **Head length (HL)	0.74 ± 0.00	0.73 ± 0.01
** Ocular diameter (MOD)**	**0.20 ± 0.00**	0.18 ± 0.00***
** **Scape length (SL)	0.51 ± 0.01	0.51 ± 0.01
**Mesosoma**		
** Mesosoma width (MW)**	**0.54** ± **0.01**	**0.39** ± 0.01***
** Mesosoma length (ML**	**1.36** ± **0.01**	**1.17** ± 0.01***
** Petiole width (PW)**	**0.31** ± **0.00**	**0.30** ± 0.00**
** Postpetiole width (PPW)**	**0.37** ± **0.00**	**0.35** ± 0.00***
** Hind femur length (HFL)**	**0.62** ± **0.01**	**0.57** ± 0.01***
**Gaster**		
** First tergum width (TW)**	**1.07** ± **0.01**	**0.96** ± 0.01***

Significant differences between the two phenotypes are given by asterisks:

* = *P* < 0.05

** = *P* < 0.01

*** = *P* < 0.001. Differences are based on tests of between-subjects effects within MANOVA. Characters in **bold font** were used in the discriminant analysis.

^**+**^ **HW**—maximum width of head immediately behind the eyes, measured in full-face view; **HL**—length of head capsule excluding mandibles, in full-face view, from the midpoint of the anterior clypeal margin to the midpoint of the posterior margin; **MOD**—maximum diameter of eye measured with the head in full lateral aspect; **SL**—maximum straight line length of scape from apex to base; **MW**—maximal width of mesosoma at the insertion of the forewing, as seen from above, at a right angle to the longitudinal axis of the mesosoma; **ML**—diagonal length of the mesosoma in profile from the anterior pronotal margin to the posterior base of the metapleural lobe; **PW**—maximum width of petiolar node, as seen from above, at a right angle to the longitudinal axis of the mesosoma; **PPW**—maximum width of postpetiole, as seen from above, at a right angle to the longitudinal axis of the mesosoma; **HFL—**length of hind femur, measured along the dorsal margin from the articulation with the trochanter to the most distal tip of the femur; **TW**—maximum width of first gastral tergum, as seen from above, at a right angle to the longitudinal axis (see S1 Dataset).

Discriminant analysis was significant (Wilks’ λ = 0.057_,_ Chi-square _8 df_ = 97.6, *P* < 0.001; Box’s M test: *F*_36, 4859_ = 1.26, *P* = 0.14), and it correctly classified 100% (40 of 40) of the queens. Again, this indicates significant morphological differences between the two phenotypes, especially given that all 40 queens were correctly classified with a probability of 1.00. The standardized canonical function coefficients indicated that mesosoma length was weighted nearly double that of all other characters (Table 2); mesosoma length was approximately 20% greater in alate queens. The second most important contributor was mesosoma width, which was approximately 33% greater in alate than in ergatoid queens.

**Table 2:** Standardized canonical discriminant function coefficients for alate and ergatoid queens of *Monomorium* sp. AZ-02.

Predictor variable	Function 1
Head width	-0.069
Maximum eye diameter	0.210
Petiole width	-0.455
Postpetiole width	-0.102
Mesosoma width	0.454
Mesosoma length	0.878
Hind femur length	0.394
First gastral tergum width	0.236

Dry mass for the two phenotypes also varied significantly with that of alate queens (1.48 ± 0.10 mg; *n* = 4 colonies) nearly twice that of ergatoid queens (0.81 ± 0.05 mg; *n* = 4 colonies; t-test, t _6 df_ = 6.2, *P* < 0.001). The low dry mass of *M*. sp. AZ-02 workers (0.046 ± 0.002 mg; *n* = 8 colonies) resulted in high queen to worker dimorphisms for both phenotypes; the dimorphism was significantly higher (t-test, t _6 df_ = 7.1, *P* < 0.001) for alate (32.5 ± 1.9) than for ergatoid queens (17.8 ± 0.8).

**Fig 1:**
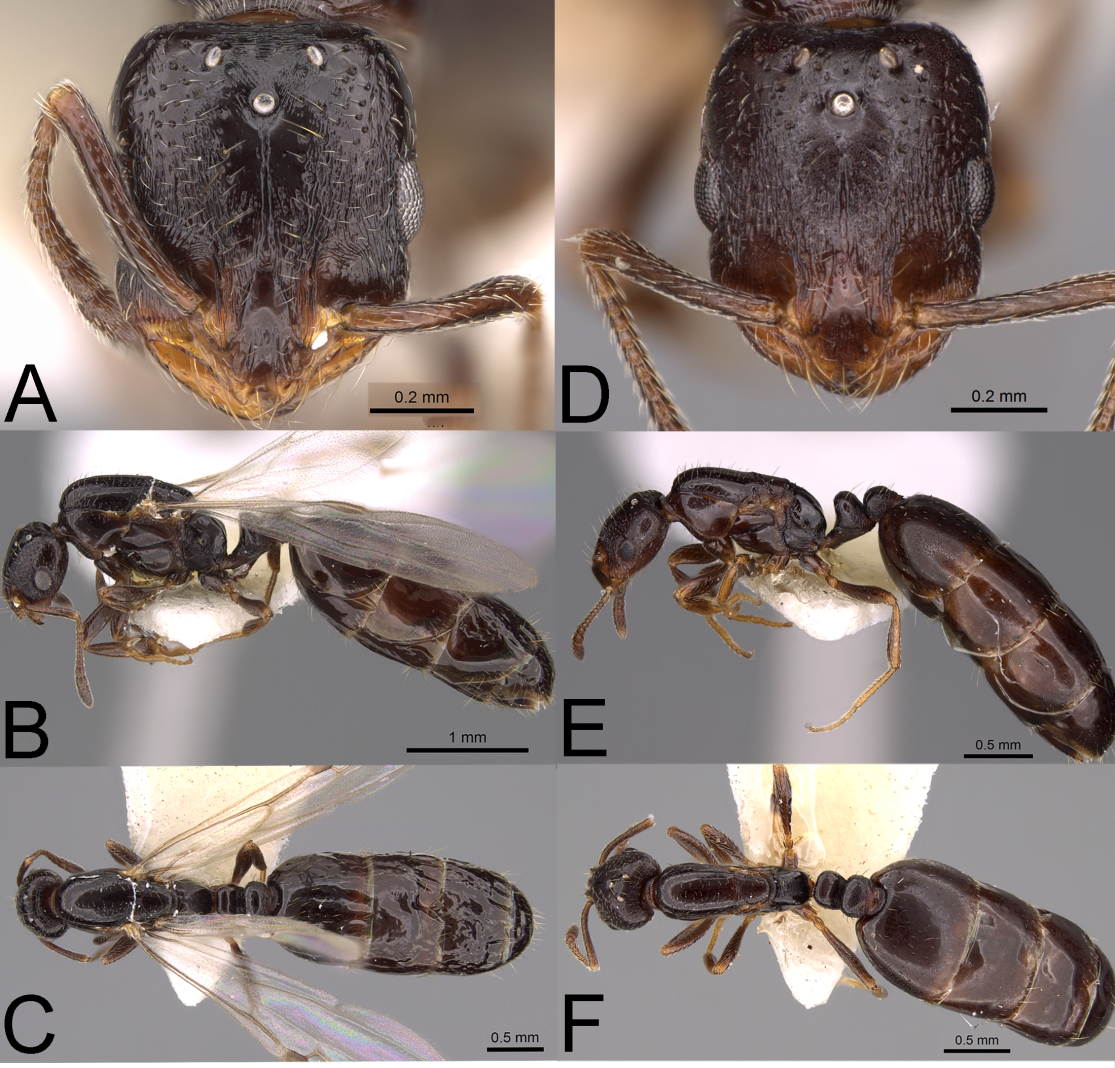
**Photographs of Monomorium sp. AZ-02 alate queen (A-C) (CASENT0914346) and ergatoid queen (D-F) (CASENT0914351). (A, D) frontal view of head, (B, E) dorsal view of body, (C, F) lateral view of body.** Photographs by Michele Esposito from www.AntWeb.org.

**Fig 2:**
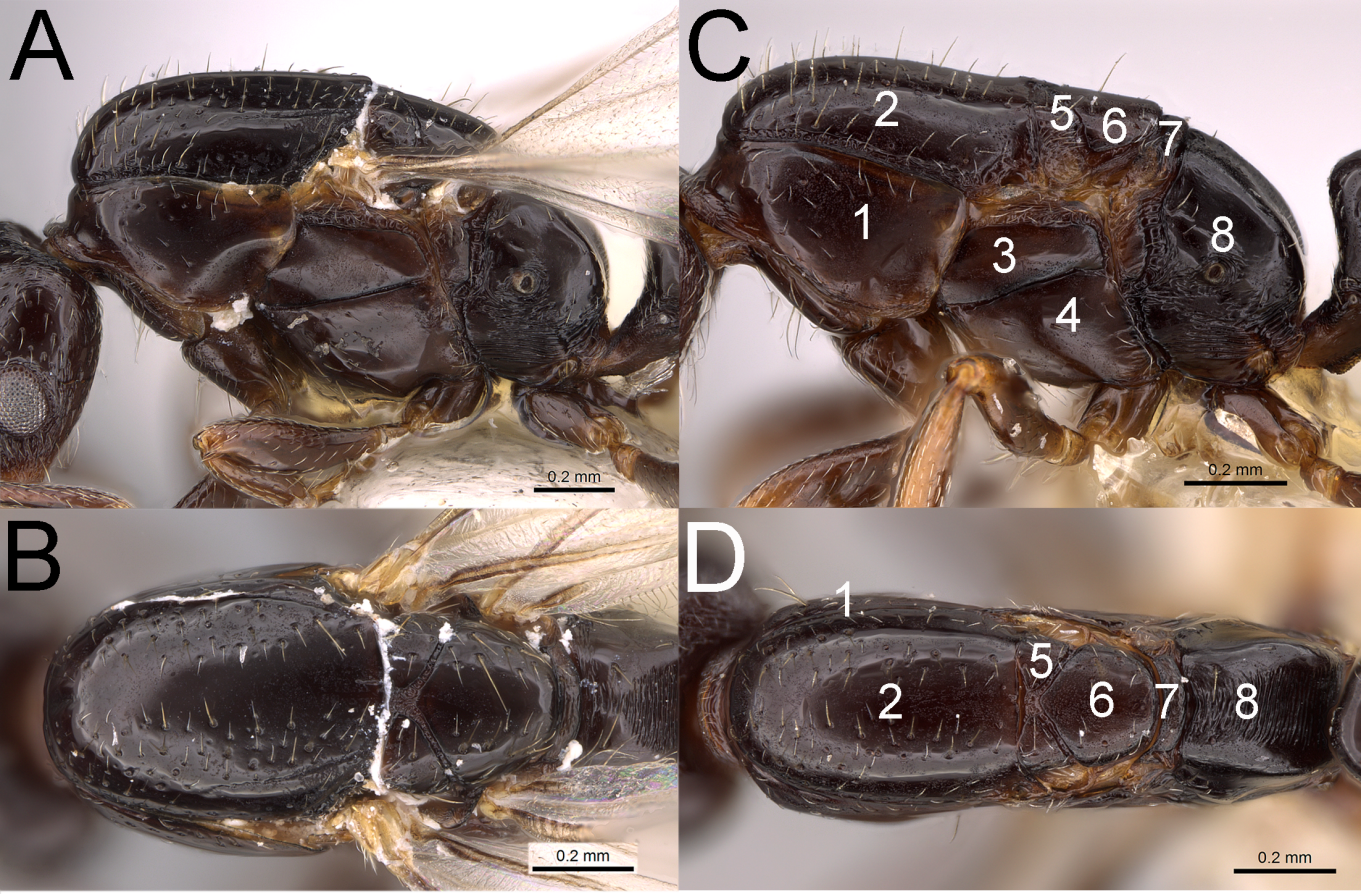
**Photographs of Monomorium sp. AZ-02 alate queen (A-B) (CASENT0914346) and ergatoid queen (C-D) (CASENT0914351). (A, C) close-up dorsal view of mesosoma, (B, D) close-up lateral view of mesosoma.** Photographs by Michele Esposito from www.AntWeb.org. Panels **C-D** show segments of mesosoma: 1 = pronotum; 2 = mesoscutum; 3 = anepisternum; 4 = katepisternum; 5 = axilla; 6 = mesoscutellum; 7 = metanotum; 8 = propodeum.

Despite size differences between alate and ergatoid queens, both phenotypes were similar in that ergatoid queens retained all mesosomal sutures and sclerites, i.e., the metanotum was not fused dorsally or laterally, the axilla was not fused to the mesoscutum, the mesoscutum and mesoscutellum were not fused, and the promesonotal suture was well-defined (see also [11]). Regardless of these similarities, the size and shape of the mesoscutum separated the two queen phenotypes (Figs 1 and 2). In alate queens (in dorsal view), the lateral and anterior margins of the mesoscutum are strongly convex and the anterior margin more broad such that the pronotum is mostly hidden below the mesoscutum; the mesoscutum also widens near the insertion for the wings. In ergatoid queens (in dorsal view), the lateral and anterior margins of the mesoscutum are narrow and subparallel, gradually narrowing toward the anterior margin, and the pronotum is enlarged and easily visible along the lateral margins of the anterior one-half of the mesoscutum; the mesoscutum also narrows near where the wings would be inserted. The pronotal profile is also diagnostic; in alate queens the pronotum is thicker (taller) and rises nearly vertically to meet the mesonotum (Fig 2A and 2B), whereas in ergatoid queens, the pronotum rises at an approximately 60° angle to meet the mesoscutum (Fig 2C and 2D). Additionally, the wing-roots of ergatoid queens are sealed by overgrowth of the cuticle. Alate and ergatoid queens also differed internally in that alate queens contained direct and indirect flight muscles while ergatoid queens lacked both sets of muscles. Both phenotypes contained phragmata (an internal ridge or projection of the body wall to which the flight muscles are attached).

### Colony structure

Occurrence of alate and ergatoid queens was highly nonrandom in this population because 43 of 43 colonies (100.0%) in which more than one queen was captured contained only one queen phenotype (*n* = 192 alate queens in 20 colonies; *n* = 126 ergatoid queens in 23 colonies). At the population level, a similar number of colonies contained alate and ergatoid queens (χ^2^_1 df_ = 0.21, *P* > 0.6). Our partial nest excavations suggested that colonies of both queen phenotypes produced a similar number of new, virgin queens (alate queen colonies: range 1–16; ergatoid queen colonies: range 1–>50)

### Lineage analysis of ergatoid and alate queens

**Fig 3:**
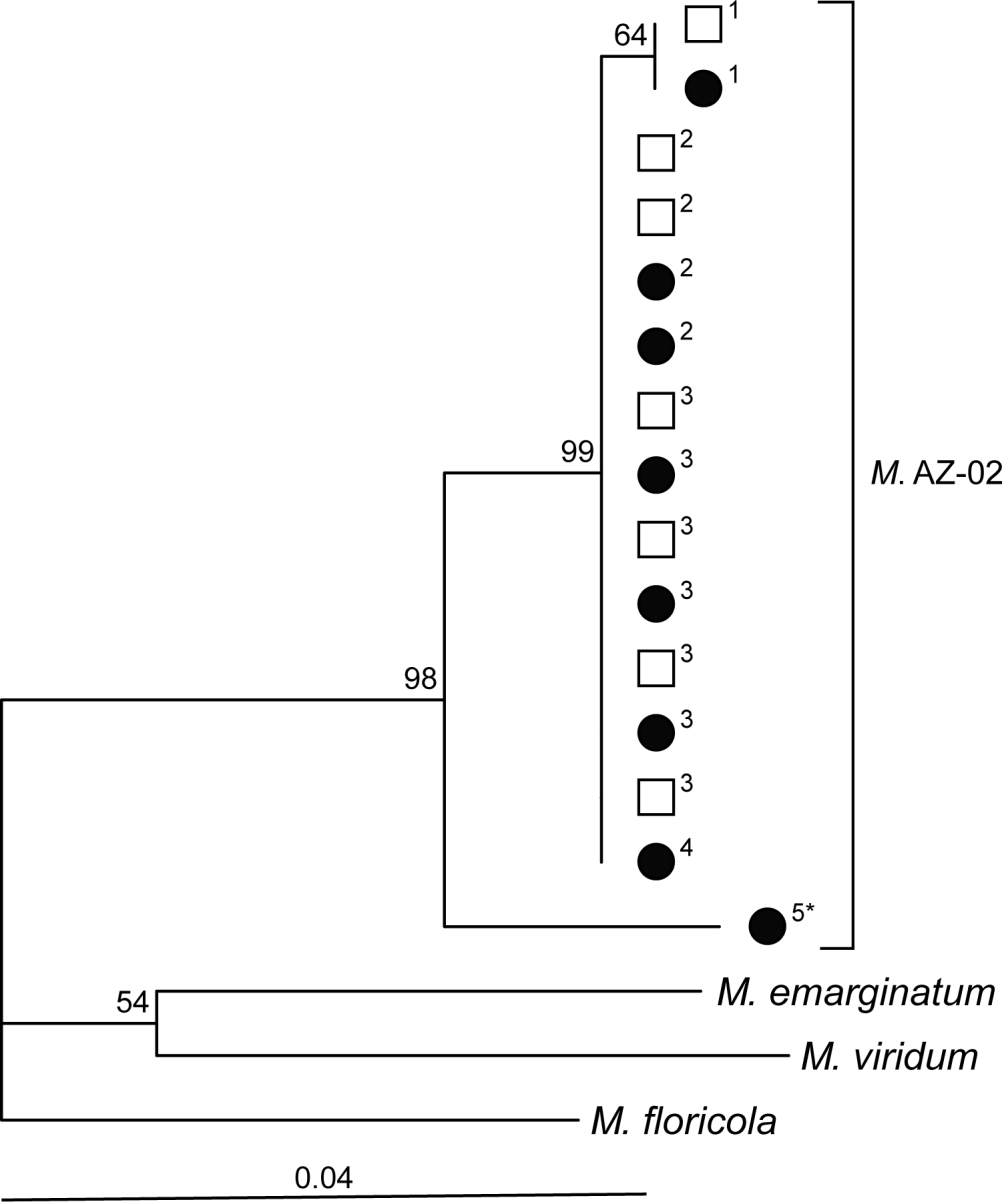
Neighbor-joining tree for alate and ergatoid queens of *Monomorium* sp. AZ-02 from our study site at Coon Bluff, Pinal County, Arizona, USA, and one sample from south of Tucson (= *); ● = alate queen; □ = ergatoid queen. The tree was reconstructed using Kimura 2-parameter pairwise distances between sequences for a 420 base pair region of the mitochondrial gene *cytochrome oxidase I*; 37 sites were parsimony informative. Bootstrap values (10,000 repetitions) are given at branch nodes. Superscripts next to phenotype symbols correspond to haplotypes; samples with the same number have the same haplotype. See text for information on outgroup samples.

The aligned matrix of 420 bp contained 344 constant sites (81.9%), 39 variable non-parsimoniously informative sites (9.3%), and 37 parsimoniously informative sites (8.8%). Intermixing of alate and ergatoid queens in our neighbor-joining tree demonstrated that the two phenotypes belong to the same mitochondrial clade (Fig 3). This conclusion is also supported by an analysis showing that sequence divergence values between the two queen phenotypes (Mann-Whitney-Wilcoxon test: mean pairwise distance ± SE = 8.00 x 10^−4^ ± 2.03 x 10^−4^) did not differ from pairwise distances within alate queens (9.34 x 10^−4^ ± 3.30 x 10^−4^, *W* = 536, *P* = 0.73) or from pairwise differences within ergatoid queens (9.34 x 10^−4^ ± 3.30 x 10^−4^, *W* = 494, *P* = 0.73). In fact, alate queens and ergatoid queens shared the same mitochondrial haplotype for three of the four haplotypes present in the Coon Bluff population (Fig 3). We found a total of five haplotypes in our 15 queen samples of *M*. sp. AZ-02.

### Mating behavior

Summer rains (during July and August) triggered mating by alate and ergatoid queens; pouring 1–1.5 l of water on nests also triggered sexuals to exit nests. Sexuals exited nests from about 0730–0900 h; ergatoid queens sometimes remained outside the nest until 1000 h, depending on the temperature. In alate queen colonies, queens flew from nests to an unknown mating location. One pair was observed on the ground *in copulo*, and one dealate and one alate foundress were located after flights, inferring that alate queens found colonies independently.

Ergatoid queens exited nests and typically climbed up stalks of vegetation, sticks, and other objects surrounding the nest, and they walked/patrolled continuously in a manner similar to that observed for workers. Ergatoid queens did not display female calling. Mating by ergatoid queens was not observed.

### Queen phenotypes in *Monomorium*

The queen caste has been described for 137 of the 358 (38.3%) described species of *Monomorium*. Queens are known for all 12 described species in the *M*. *minimum*-group in North America; five of these species have only alate queens (41.7%), three species have only ergatoid queens (25.0%), and four species have alate and ergatoid queens (33.3%); four additional undescribed species include two species with ergatoid queens and two species with alate and ergatoid queens (Table 3). Worker-queen intercastes or worker-queen intergrades are not known to occur in the *M*. *minimum*-group [31]. Thirty-five of the remaining 125 species (28.0%) have queens that are wingless (ergatoid, brachypterous); 15 of these 35 species (42.9%) also have alate queens (Table 4). The species identity is unknown for two additional Australian species (*Chelaner* sp. and *M*. sp. 10) [14, 32] that have alate and brachypterous/ergatoid queens (Table 4). Overall, wingless queen species of *Monomorium* are geographically and taxonomically widespread as they occur on several continents (Africa, Asia, Australia, Europe, North America) and in eight species groups (*bicorne*, *kiliani*, *longinode*, *minimum*, *monomorium*, *orientale*, *rubriceps*, *salomonis*) (Table 4).

**Table 3:** Known queen phenotypes for *Monomorium minimum*-group species in North America; species are listed alphabetically. For queen phenotype: EQ = ergatoid queen; AQ = alate queen. Taxonomy follows Bolton [33].

Species	Queen phenotype	References
*compressum* Wheeler	EQ	[31, 34]
*cyaneum* Wheeler	EQ	[31, 34, 35]
*ebeninum* Forel	EQ	[31, 36]
*emarginatum* DuBois	AQ	[31]
*ergatogyna* Wheeler	AQ + EQ	[31, 36, 37]; P.S. Ward, pers. comm.
*inquilinum* DuBois*	AQ	[31, 35]
*marjoriae* DuBois	AQ	[31]
*minimum* (Buckley)	AQ + EQ	[31, 38]; [39, as *M*. *metoecus*]
*pergandei* (Emery)*	AQ	[31, 35]; [40, as *Epoecus pergandei*]
*talbotae* DuBois*	AQ	[31, 35]
*trageri* DuBois	AQ + EQ	[31]
*viridum* Brown	AQ + EQ	[31, 41]; [42, as *M*. *peninsulatum*]
sp. AZ-01	AQ + EQ	S.P. Cover, pers. comm.; R.A. Johnson, pers. obs.
sp. AZ-02	AQ + EQ	this study
sp. AZ-03	AQ + EQ	S.P. Cover, pers. comm.; R.A. Johnson, pers. obs.
sp. cf. *ergatogyna*	EQ	P.S. Ward, pers. comm.; R.A. Johnson, pers. obs.

* denotes inquiline species

**Table 4:** Species of *Monomorium* outside of North America that have ergatoid queens (EQ) or brachypterous (= non-functional, short-winged) queens (BQ); alate queens (AQ) also occur in some of these species.

Species	Type locality (country)	Queen phenotype	References
***M*. *bicorne-*group**			
*striatifrons* Heterick	AUSTRALIA	AQ + BQ	[28]
***M*. *kiliani-*group**			
*tambourinense* Forel	AUSTRALIA	AQ + EQ (EQ or WQI)	[28, 43]; [44, as *M*. *howense*]
***M*. *longinode-*group**			
*flavonigrum* Heterick	AUSTRALIA	EQ	[28]
***M*. *monomorium-*group**			
*bogischi* Wheeler	AUSTRALIA	BQ	[10, 45]
*castaneum* Heterick	AUSTRALIA	AQ + BQ (EQ or WQI)	[28]
*floricola* (Jerdon)	INDIA	AQ + BQ + EQ	[10, 29, 36, 46–49]
*laeve* Mayr	AUSTRALIA	AQ + BQ	[28]
*rothsteini* Forel	AUSTRALIA	AQ + BQ	[28]
*sordidum* Forel	AUSTRALIA	AQ + BQ	[28]; [50, as *M*. *micron*]
*subapterum* Wheeler	AUSTRALIA	BQ	[10, 45]
** sp.^+^	AUSTRALIA	AQ + BQ	[14, as *Chelaner* sp.]
***M*. *orientale-*group**			
*orientale* Mayr	INDIA	EQ	[46]
***M*. *rubriceps-*group**			
*leae* Forel	AUSTRALIA	AQ + EQ (EQ or WQI)	[10, 28, 51]
*rubriceps* Mayr	AUSTRALIA	AQ + EQ (EQ or WQI)	[10, 28, 52]
** cf. *rubriceps*	AUSTRALIA	AQ + EQ	[27]
***M*. *salomonis-*group**			
*advena* (Brown & Wilson)	ISRAEL	EQ	[11, 26]; [53, as *Epixenus andrei* Emery]
*albopilosum* Emery	SOUTH AFRICA	AQ + EQ	[11, 26]; B. Bolton, pers. comm.
*algiricum* (Bernard)	ALGERIA	EQ	[11, 26, 54]; [55, as *Epixenus algiricus*]
*andrei* Saunders	GIBRALTAR	AQ + EQ	[54]; [56, as *M*. *andrei fur*]
*bicolor* Emery	ETHIOPIA	AQ + EQ	[26, 57]; B. Bolton, pers. comm.
*biroi* Forel	INDIA	EQ	[11, 26]
*boltoni* Espadaler & Agosti	CAPE VERDE ISLAND	EQ	[58]
*damarense* Forel	NAMIBIA	EQ	[26]
*dichroum* Forel	INDIA	AQ + EQ	[10, 11, 26]
*grassei* (Tohmé & Tohmé)	SYRIA	EQ	[11, 26]; [53, as *Epixenus grassei*]
*hesperium* (Emery)	CANARY ISLANDS	EQ	[11, 26, 59]
*libanicum* (Tohmé & Tohmé)	LEBANON	EQ	[11, 26]; [53, as *Epixenus libanicus*]
*medinae* Forel	TENERIFE	EQ	[11, 26]; [60, as *Xenhyboma mystes*]
*minor* Stitz	NAMIBIA	EQ	[11, 26]
*opacior* Bolton	ZIMBABWE	EQ	[11, 26, 61]
*rufulum* Stitz	NAMIBIA	AQ + EQ	[11, 26]
*sahlbergi* Emery	ISRAEL	EQ	[49]
*syriacum* (Tohmé & Tohmé)	SYRIA	EQ	[11, 26]; [53, as *Epixenus syriaca*]
*venustrum* (Smith)	SYRIA	AQ + EQ	[10, 11, 49, 62, 63]
*wilsoni* Espadaler	CANARY ISLANDS	EQ	[64]
**Unplaced species**			
*creticum* Emery	GREECE	EQ	[65, 66]
*schurri* Forel	INDIA	EQ	[10, 67]

Species are listed alphabetically by species group and species within a group. Taxonomy follows Bolton [33]. WQI = worker-queen intercaste or worker-queen intergrade (see text).

^**+ **^Heterick [28] placed this species in the *M*. *rothsteini*-group, but did not identify the species. Thus, it may be a species already on the list or another described or undescribed species.

An additional five species have ergatoid queens and/or WFI, but definitive information is lacking (Table 5). Two other species are included in this group because it is unclear if the description was that of a worker or queen (*M*. *sichelii*) or because the identity is unclear (Table 5).

**Table 5:** Species of *Monomorium* outside of North America that have ergatoid queens and/or worker-queen intercastes or worker-queen intergrades (WQI) (in parentheses).

Species	Type locality (country)	Queen phenotype	References
***M*. *falcatum-*group**			
* falcatum* (McAreavey)	AUSTRALIA	WFI	[30, as *Schizopelta falcata*]
***M*. *monomorium-*group**			
* exiguum* Forel	ETHIOPIA	AQ + (EQ or WQI)	[29]
* termitobeum* Forel	MADAGASCAR	AQ + (EQ or WQI)	[29]; [68, as *M*. *imerinense*]
***M*. *rubriceps-*group**			
* centrale* Forel	AUSTRALIA	AQ + (EQ or WQI)	[28]
* sculpturatum* Clark	AUSTRALIA	AQ + (EQ or WQI)	[28, 69]
***M*. *salomonis-*group**			
* sichelii* (Roger)^#^	SPAIN	EQ	[26]; [70, as *Phacota sichelii*]
**Unplaced species**			
* * sp. 10*	AUSTRALIA	AQ + EQ	[32]

No more information was given in the publications, so it is unknown which phenotype was examined. These species are included to emphasize that the queen phenotypes need to be examined in more detail. *Monomorium* sp. 10 is placed in this table because its identity is unclear (see below). Species are listed alphabetically by species group and species within a group. Taxonomy follows Bolton [33].

^**# **^Bolton [26] discusses the description of *M*. *sichelli* holotype worker, and suggests that this specimen may have been an ergatoid queen. However, the specimen is presumed lost and the species has not been found again.

## Discussion

### Evolution of queen dimorphism

Dispersal polymorphisms occur in a wide variety of insects, and they are particularly well known in crickets and aphids [1, 71]. Intraspecific queen dimorphisms in the form of winged and wingless queens also are relatively common in ants, with examples scattered across genera in several subfamilies [3, 8], and additional queen dimorphic ant species are documented regularly, to the point that these polyphenisms should be viewed as a common and regular life history strategy for ants (see also [72–73]). Our genetic data document queen dimorphism in *M*. sp. AZ-02, while our survey data across species of *Monomorium* indicate that numerous congeners on several continents and in several species groups also have dimorphic queens. Numerous additional species are thusfar known to have only ergatoid or brachypterous queens (Tables 4 and 5). It should also be noted that numerous queen descriptions were based on dealate queens (after the wings are shed), and that dealate brachypterous queens are easily mistaken for fully winged queens that have dealated (see [8]). That these wingless queen species are widespread geographically and taxonomically suggests that winglessness has evolved multiple times, possibly as a secondary modification in response to local environmental conditions [2, 3]. A phylogeny of the *Monomorium* and sister genera is necessary to determine the ancestral state for the queen phenotype. Appearance of this trait in several species groups and on several continents suggests that the genetic potential for ergatoid and/or brachypterous queens may be present in a large number of species, similar to the expression of polymorphism in trimorphic species of *Pheidole* [74].

Data on queen phenotype are meager for most species listed in Tables 3–5. For example, Heterick [28, 29] provided information on queen phenotype for species from Australia and Madagascar, but said only that he examined brachypterous or ergatoid queens (or worker-female intercastes). He did not give information on their number or frequency relative to alate queens. More detailed information was given for only *M*. *sordidum*, where it was indicated that brachypterous queens were common [28]. Additional collecting, combined with a taxonomic revision that includes molecular genetic analyses, will likely document that more of these species possess dimorphic queens, especially given that queens typically are known only from one to few colonies. Queens also should be collected from multiple colonies and/or multiple locales because one phenotype may be rare, as occurs for alate queens in *M*. *ergatogyna* (P.S. Ward, pers. comm.) and *M*. sp. AZ-01 (S.P. Cover, pers. comm.).

One caveat to this discussion is to note that *Monomorium* remains taxonomically unstable. As noted by Ward et al. [75], *Monomorium* remains a heterogenous, non-monophyletic genus. A number of species have recently been moved to different genera (e.g., *Syllophopsis*, *Trichomyrmex*, *Royidris*, *Epelysidris*) [75], and future molecular genetic studies will likely move additional species to other genera. Regardless, this discussion emphasizes that *Monomorium* (and possibly closely related genera) is an ideal genus to examine evolution and development of queen phenotypes because ergatoid and brachypterous queen species are common.

In North America, queen dimorphic species in the *M*. *minimum*-group are geographically diverse as they occur in habitats that range from hot desert to alpine forests. Some of these species are ideal for studying evolution and development because they occur in multiple, isolated, relict populations in the high-elevation “sky islands” of the southwestern United States and northwestern Mexico. These “islands” have been isolated for 10,000–20,000 years, and they have undergone several rounds of glaciation and retreat [76], such that they are natural replicates for studying evolution of wing development (see [12]). Additionally, queenright colonies of these species are relatively easy to capture and maintain, and laboratory colonies produce sexuals ([12], R.A. Johnson, pers. obs.).

We were not able to excavate the reproductive queen(s) from any colonies of *M*. sp. AZ-02. Consequently, we cannot infer the mechanism that determines queen phenotype. However, that nests with each queen phenotype occur sympatrically at a similar frequency suggests that phenotype is not related to the abiotic environment. Other potential mechanisms include maternal effects, direct or indirect pheromonal effects of the queen, or nuclear genetic factors. That different species of *Monomorium* have different patterns of queen occurrence in nests suggests that multiple mechanisms determine queen phenotype in this genus. These patterns include: (1) species in which colonies produce only one queen phenotype (*M*. sp. AZ-02, this study), (2) species that have dealate and ergatoid queens in one nest simultaneously (*M*. sp. AZ-03, R.A. Johnson, pers. obs.), (3) species that have both phenotypes in one nest, but not at the same time (*M*. cf. *rubriceps* [27]), and (4) species in which alate queens produce alate queens, whereas ergatoid queens produce alate and ergatoid queens (*M*. sp. 10 [32]).

### Alternative life histories

Dispersal polyphenisms in ants typically are associated with different methods of colony founding. Dealate queens display independent colony founding, while ergatoid queens display dependent colony founding. In the latter case, ergatoid queens mate, return to the nest, then leave with a retinue of workers to start a nest at a later date [8, 77]. These different life histories are viewed as mechanisms to disperse to distant or isolated habitats (alate queens) or to move short distances within homogenous habitats (ergatoid queens) [2, 4]. *Monomorium* sp. AZ-02 appears to be restricted to occurring in shaded to semi-shaded microsites under tree canopies. These microhabitats represent large, mostly contiguous habitats at the study site, whereas in open, exposed, Sonoran Desert habitats, clumps of one to few trees represent small, isolated habitats that likely are difficult for ergatoid queens to colonize.

Queens of some species choose not to fly despite the presence of fully-developed, functional wings, including *Monomorium pharaonis* in which queens mate and shed their wings inside their natal colony [8, 11]. Our observations of alate queens of *M*. sp. AZ-02 flying from the nest document that this species has a dispersal polymorphism given that ergatoid queens can only return to their natal nest or disperse on foot.

### Morphometrics

This is the first study to conduct a morphometric comparison for a queen dimorphic species of *Monomorium*. Our mass data for *Monomorium* sp. AZ-02 demonstrate the large size dimorphism between both queen phenotypes and workers (alate queen: worker dry mass = 32.5; ergatoid queen: worker dry mass ratio = 17.8). This large size dimorphism is uncommon in ants, and it also occurs in other species of *Monomorium*, *Onychomyrmex*, and the dorylines [see 8]. Like the dorylines, species of *Onychomyrmex* have an army ant lifestyle that includes group predation and nomadism. That ergatoid queens for least some species of *Monomorium* have a large queen to worker size dimorphism suggests that this trait may likewise be related to some aspect of their life history. The significant difference in dry mass between alate and ergatoid queens resulted from the larger mesosoma and accompanying flight muscles possessed by alate queens (see Fig 2B and 2D). *Monomorium* sp. AZ-02 also presents an example of ergatoid queens possessing internal phragmata, which are cuticular structures that develop during the pupal stage and are fixed throughout adult life. Their presence may indicate that wing muscles develop in part and are histolyzed post-emergence given that occasional ergatoid pupae/adults have wing muscles in congeners (e.g., *M*. sp. 10 [32]).

The mesosoma is the primary character to distinguish between alate and ergatoid queens of *M*. sp. AZ-02 (except for presence of wings). Consequently, careful examination of the mesosoma is needed to distinguish between dealate and ergatoid queens. These similarities include that ergatoid queens: (1) retain all flight sclerites possessed by alate queens, (2) their wing-roots are sealed by an overgrowth of cuticle, and (3) the dorsal outline of the mesoscutum is slightly concave to saddle-shaped. That their mesosomal morphology is similar to that of alate queens suggests that ergatoid queens of *M*. sp. AZ-02 are derived recently, especially considering the morphological reductions in size and number of mesosomal sclerites for many congeneric ergatoid queens (see [11]).

In conclusion, the ant genus *Monomorium* is ideal for examining development and evolution of queen phenotypes given that wingless queens (ergatoid, brachypterous) occur in approximately 30% (42 of 137) of the species in which queens have been described. Given this high percentage, numerous other wingless queen species undoubtedly occur in the genus, both in species where the queen is unknown and in unknown species. The first step to advance our knowledge in this respect is to make numerous collections and conduct field studies on species listed in Tables 3–5. Such information would include: (1) molecular genetic analyses to document that dimorphic queens belong to the same gene pool, that there is unrestricted gene flow between the two queen forms, and that there is an absence of assortative mating and inbreeding by males (see [73], this study), (2) comparative internal and external morphology for queen dimorphic species, (3) determine if both queen phenotypes occur in sympatry for queen dimorphic species, and if both phenotypes are produced as a regular part of the colony life cycle, (4) determine if queen dimorphic species produce these two phenotypes simultaneously or sequentially, (5) laboratory studies that cross-foster sexual brood so as to understand the mechanism(s) determining queen phenotype, and (6) mapping queen phenotypes onto a phylogeny of the genus.

## Supporting information

S1 Dataset (XLSX)
